# Clinicopathological features, genetic alterations, and *BRCA1* promoter methylation in Japanese male patients with breast cancer

**DOI:** 10.1007/s10549-022-06822-x

**Published:** 2022-12-09

**Authors:** Akihiko Shimomura, Masayuki Yoshida, Takashi Kubo, Satoshi Yamashita, Emi Noguchi, Aiko Nagayama, Toru Hanamura, Miki Okazaki, Toru Mukohara, Asako Tsuruga, Kiyo Tanaka, Yukino Kawamura, Toru Higuchi, Yoko Takahashi, Sasagu Kurozumi, Tetsu Hayashida, Hitoshi Ichikawa, Toshikazu Ushijima, Akihiko Suto

**Affiliations:** 1 Department of Breast and Medical Oncology, National Center for Global Health and Medicine, 1-21-1 Toyama, Shinjuku-Ku, Tokyo, 162-8655 Japan; 2 Department of Diagnostic Pathology, National Cancer Center Hospital, Tokyo, Japan; 3 Department of Clinical Genomics, National Cancer Center Research Institute, Tokyo, Japan; 4 Division of Epigenomics, National Cancer Center Research Institute, Tokyo, Japan; 5 Department of Medical Oncology, National Cancer Center Hospital, Tokyo, Japan; 6 Department of Surgery, Keio University School of Medicine, Tokyo, Japan; 7 Department of Breast Oncology, Tokai University School of Medicine, Kanagawa, Japan; 8 Department of Breast Oncology, Tokyo Medical University, Tokyo, Japan; 9 Department of Medical Oncology, National Cancer Center Hospital East, Kashiwa, Japan; 10 Division of Breast Surgical Oncology, Department of Surgery, Showa University Hospital, Tokyo, Japan; 11 Department of Breast and Endocrine Surgery, Toranomon Hospital, Tokyo, Japan; 12 National Center for Global Health and Medicine Research Course in Advanced Medical Specialties, Juntendo University Cooperative Graduate School, Tokyo, Japan; 13 Breast Unit, Japanese Red Cross Saitama Hospital, Saitama, Japan; 14 Department of Breast Surgical Oncology, Cancer Institute Hospital of Japanese Foundation for Cancer Research, Tokyo, Japan; 15 Department of Breast Surgery, International University of Health and Welfare, Chiba, Japan; 16 Department of Breast Surgery, National Cancer Center Hospital, Tokyo, Japan

**Keywords:** Male breast cancer, Rare cancers, Descriptive studies, Translational research, Biomarkers

## Abstract

**Purpose:**

Male breast cancer (MBC) is a rare cancer accounting for only 1% of all male cancers and is, therefore, poorly studied. We aimed to characterize the subtypes of MBC in Japanese patients based on genetic profiling, the presence of tumor-infiltrating cells, and the expression of immunohistochemical markers.

**Methods:**

This retrospective study included 103 patients with MBC diagnosed between January 2009 and December 2019 at various hospitals in Japan. Clinicopathological patient characteristics were obtained from medical records, and formalin-fixed paraffin-embedded tissue specimens were analyzed for histological markers, mutations of 126 genes, *BRCA1* methylation, and stromal tumor-infiltrating lymphocytes.

**Results:**

The median patient age was 71 (range 31–92) years. T1-stage tumors were the most frequent (47.6%), and most were node negative (77.7%). The majority of tumors were positive for estrogen receptor (98.1%), progesterone receptor (95.1%), and androgen receptor (96.1%), and *BRCA2* was the most frequently mutated gene (12.6%). The most common treatment was surgery (99.0%), either total mastectomy (91.1%) or partial mastectomy (7.0%). Survival analysis showed a 5-year recurrence-free survival rate of 64.4% (95% confidence interval [CI] 46.7–88.8) and a 5-year overall survival rate of 54.3% (95% CI 24.1–100.0).

**Conclusion:**

Japanese MBC is characterized by a high rate of hormonal receptor positivity and *BRCA2* somatic mutation. Due to the observed clinicopathological differences in MBC between the Western countries and Japan, further prospective studies are needed to evaluate the most suitable treatment strategies.

**Supplementary Information:**

The online version contains supplementary material available at 10.1007/s10549-022-06822-x.

## Introduction

Male breast cancer (MBC) is rare, estimated to account for only 1% of all breast cancers. Consequently, it is poorly studied, with a lack of prospective clinical trials that evaluate treatment outcomes. Recent studies have shown that molecular expression patterns in MBC differ from those in female breast cancer (FBC), with a frequent expression of (hormone receptors [HRs]; estrogen receptor [ER], progesterone receptor [PgR], and androgen receptor [AR]) and greater sensitivity to hormonal therapies [1–4]. Although MBC and FBC differ in biology and genetic risk factors [5], they are generally treated in the same way [6].

MBC is usually diagnosed at an older age compared to FBC (68 vs. 62 years) [7], and patients tend to have a more advanced disease stage at diagnosis [5, 8, 9]. Five-year overall survival is significantly lower in patients with MBC than in patients with FBC (45.8% vs. 60.4%), probably due to differences in disease pathogenesis and pathophysiology, treatment compliance rates, lifestyle factors, or lack of favorable treatment outcomes for MBC [10].

Several recent studies have attempted to characterize MBC in Western countries (the US and Europe); however, there have been rare previous studies on Asian patients with MBC. The incidence, survival rate, and molecular expression of a cancer, as well as response to therapy, may differ significantly between populations of different races; however, the majority of patients included in clinical studies are Caucasians. Asians make up 60% of the world population, but only 5% of clinical trial participants are Asians. Failure to account for racial disparities in clinical trials may result in corresponding disparities in the quality of health care received [11].

Although MBC is considered rare, 129 Japanese men died of it in 2020 [12]. To ensure appropriate treatment of Japanese patients with MBC, there is a need to fully clinically characterize MBC subtypes in this population and to conduct prospective clinical trials to evaluate treatment outcomes. As a first step toward this goal, we aimed to characterize MBC subtypes in Japanese patients based on genetic profiling, as well as to determine the presence of tumor-infiltrating lymphocytes (TILs) and the expression of immunohistochemical markers such as ER, PgR, and human epidermal growth factor receptor 2 (HER2).

## Methods

### Patient data and sample collection

This retrospective study included patients diagnosed with MBC between January 2009 and December 2019 at the following participating institutions in Japan: National Center for Global Health and Medicine; National Cancer Center Hospital; Keio University School of Medicine; Tokai University Hospital; Tokyo Medical University; National Cancer Center Hospital East; Showa University Hospital; Toranomon Hospital; Japan Red Cross Saitama Hospital; Cancer Institute Hospital; International University of Health and Welfare Hospital. All patients for whom medical records and tissue samples were available were considered for the study. The patients’ medical records were used to obtain clinicopathological data at baseline and follow-up, including patient characteristics, tumor stage, tumor histology, treatments used (systemic or otherwise), and other information. Tumor stage was assessed based on the TNM Classification of Malignant Tumors, 8th edition [13]. Tissue specimens were obtained from primary or metastatic tumors at the time of surgery or biopsy and processed into formalin-fixed paraffin-embedded (FFPE) blocks. Patients were included in the study if sufficient FFPE samples of their tumors were available for analysis. Each sample was evaluated by an expert pathologist (MY). This study was performed in line with the principles of the Declaration of Helsinki. Approval was granted by the Institutional Review Board of the National Center for Global Health and Medicine, Tokyo (Date: March 20, 2020; No. NCGM-G-003481-01). The requirement for informed consent to participate was waived because of the retrospective nature of the study.

### Stromal TIL assessment

Tumor sections were stained with hematoxylin and eosin, and the proportion of TILs in the sections was assessed according to the recommendations of the International TILs Working Group [14]. TILs in the tumor stroma and outside the tumor cell nests were considered stromal TILs (sTILs), and sTIL percentage was estimated as the proportion of the stroma occupied by sTILs.

### Immunohistochemistry

We evaluated the expression of ER, PgR, HER2, androgen receptor (AR), and programmed death-ligand 1 (PD-L1). Immunohistochemical staining methods are shown in Table S1. ER and PgR levels were evaluated using the Allred scoring system [15], and a proportion score of 2 or above (> 1% nuclear-stained tumor cells) was considered positive, following the guidelines of the American Society of Clinical Oncology and College of American Pathologists (ASCO/CAP) [16]. HER2 status was determined according to ASCO/CAP guidelines [17]. The expression of AR was evaluated using the same method used for ER and PgR. PD-L1 score (IC0–IC2) was based on the proportion of the tumor area occupied by PD-L1–expressing immune cells, and IC1 or above (≥ 1%) was considered positive [18]. The Ki-67 labeling index (the proportion of Ki-67–positive cells) was determined by visual counting of 500 cancer cells in a hotspot.

### Gene alteration measurement

To analyze gene alterations, we used the NCC Oncopanel test (v. 4), which can detect mutations and copy number alterations of 114 genes together with rearrangements of 12 oncogenes [19]. Genomic DNA was extracted from 10 4-μm sections of FFPE blocks using the QIAamp DNA FFPE Tissue Kit (Qiagen #56404, Hilden, Germany). The extracted DNA was quantified using a Qubit dsDNA BR Assay Kit (Thermo Fisher Scientific #Q32850, Waltham, MA, USA) and a Qubit 3.0 Fluorometer (Thermo Fisher Scientific #Q33216). The pathogenicity of the identified gene alterations was determined using public databases (COSMIC and ClinVar). Gene alterations were considered pathogenic if they were registered as pathogenic or likely pathogenic in ClinVar; if they were a splicing-site, stop-gain, or frameshift variant in a tumor suppressor gene; or if they were registered five or more times in COSMIC as confirmed somatic variants. Variants were excluded if they were suspected to be germline single-nucleotide pleomorphisms (SNPs) based on variant allele frequency (VAF) and were registered with more than 1% frequency in databases of single-nucleotide polymorphisms: 1000 Genomes (1 kgp, 201,204) (http://ww- w.1000genomes.org); the NHLBI GO Exome Sequencing Project (ESP6500) (http://evs.gs.washington.edu/EVS/); the Human Genetic Variation Database (HGVD, 20,131,010) (http://www.genome. med.kyoto-u.ac.jp/SnpDB); and the Integrative Japanese Genome Variation Database (iJGVD, 20,151,218) (https://ijgvd.megabank. tohoku.ac.jp/) [19] or if VAF was < 5% (indicating possible DNA contamination).

### Methylation analysis

Methylation analysis was performed as previously described [20] using 500 ng of genomic DNA and the EZ DNA Methylation Kit (Zymo Research D5002, Irvine, CA, USA). The methylation level was calculated as a percentage of the level of a methylated reference DNA standard containing 10^1^–10^6^ molecules, then normalized to the same measurement obtained using *Sss*I-treated genomic DNA. This calculation was performed as [(number of methylated DNA fragments in sample)/(number of methylated and unmethylated DNA fragments in samples)]/[(number of methylated DNA fragments in *Sss*I-treated DNA)/(number of methylated and unmethylated DNA fragments in *Sss*I-treated DNA)] × 100.

### Statistical analysis

Recurrence-free survival (RFS) and overall survival (OS) rates were calculated using the Kaplan–Meier method. All statistical analyses were performed using R software (v. 4.0.4).

## Results

### Patient characteristics

A total of 103 patients were included in this study. Patient demographics are shown in Table 1. The median age was 71 (range 31–92) years. The most frequently observed tumor stages were T1 (47.6%), N0 (node-negative; 77.7%), and M0 (95.1%). The most common treatment was surgery (99.0%), either total mastectomy (91.1%) or partial mastectomy (7.0%). Only one patient underwent systemic therapy instead of surgery, due to the presence of concomitant malignancies (lung and liver cancer). Axillary surgery with sentinel node biopsy was performed in 60.8% of patients, and axillary dissection was performed in 32.4%. Surgery was accompanied by neoadjuvant systemic treatment in 11.7% of patients, adjuvant systemic therapy in 95.1%, and radiotherapy in 12.7%.

**Table 1:** Clinicopathological characteristics of patients with MBC included in the study (*N* = 103)

Age, median (range)			71 (31–92)
TNM,* n* (%)	T	Tis	4 (3.9)
		T1	49 (47.6)
		T2	31 (30.1)
		T3	3 (2.9)
		T4	13 (12.6)
		Unknown	3 (2.9)
	N	N0	80 (77.7)
		N1	18 (17.5)
		N2	0 (0)
		N3	3 (2.9)
		Unknown	2 (1.9)
	M	M0	98 (95.1)
		M1	2 (1.9)
		Unknown	3 (2.9)
Surgery,* n* (%)	Yes		102 (99)
	No		1 (1.0)
Surgical procedure,* n* (%)	Bp		2 (2.0)
	Bp + Snb		1 (1.0)
	Bp + ALND		4 (4.0)
	Bt		3 (2.9)
	Bt + Snb		61 (59.8)
	Bt + ALND		29 (28.4)
	Unknown		2 (2.0)
Systemic treatment,* n* (%)	Neoadjuvant	Yes	12 (11.7)
		No	91 (88.3)
	Adjuvant	Yes	98 (95.1)
		No	5 (4.9)
	Radiotherapy	Yes	13 (12.7)
		No	87 (85.3)
		Unknown	2 (2.0)
Systemic drug treatment,* n* (%)	Cytotoxic chemotherapy	Yes	31 (30.1)
		No	72 (69.9)
	Endocrine therapy	Yes	95 (92.2)
		No	8 (7.8)
	Anti-HER2 therapy	Yes	5 (4.9)
		No	98 (95.1)
Comorbidity,* n* (%)	Yes		65 (63.7)
	No		38 (36.3)
		Hypertension	46 (44.7)
		Other malignancy	33 (32)
		Coronary disease	13 (12.6)
		Diabetes	11 (10.7)
		Cerebrovascular disease	9 (8.7)
		Hepatitis	5 (4.9)
		Renal disease	4 (3.9)
		CHF	2 (1.9)
		COPD	2 (1.9)
		Collagen disease	0 (0)
Family history of cancer,* n* (%)	Yes		51 (49.5)
	No		47 (45.6)
	Unknown		5 (4.9)
		Breast	24 (23.3)
		Colorectal	13 (12.6)
		Gastric	8 (7.8)
		Pancreas	4 (3.9)
		Prostate	1 (1.0)
		Ovary	0 (0)
		Others	23 (22.3)

*Bp* partial mastectomy, *SNB* sentinel lymph node biopsy, *ALND* axillary lymph node dissection, *Bt* total mastectomy, *CHF* congestive heart failure, *COPD* chronic obstructive pulmonary disease, *TNM*, TNM Classification of Malignant Tumors, 8th edition

Most patients with MBC (63.7%) had comorbidities, including hypertension (44.7%), coronary artery disease (12.6%), diabetes (10.7%), cerebrovascular disease (8.7%), hepatitis (4.9%), and renal disease (3.9%), as well as other cancers, of which gastric cancer (7.8%) was the most common, followed by colorectal cancer (6.8%), prostate cancer (6.8%), esophageal cancer (2.9%), and pharyngeal cancer (1.9%) (Table S2).

Approximately half of the patients with MBC (49.5%) reported a family history of cancer including breast cancer (23.3%), colorectal cancer (12.6%), gastric cancer (7.8%), pancreatic cancer (3.9%), or prostate cancer (1.0%). None reported a family history of ovarian cancer.

### Tumor characteristics

We performed an immunohistochemical analysis of the expression of the breast cancer markers ER, PgR, HER2, and PD-L1 in the FFPE specimens. The majority of tumors were ER + (98.1%), PgR + (95.1%), and AR + (96.1%); thus, most tumors were HR + , whereas HER2 positivity was observed in only 6.8% of patients. The majority of the specimens exhibited < 10% sTILs. The Ki-67 labeling index tended to be low (mean ± SD, 17.55 ± 18.17) (Table 2).

**Table 2:** Marker characteristics of analyzed tumors (*N* = 103)

Marker		*n* (%)
ER, *n* (%)	Positive	101 (98.1)
	Negative	2 (1.9)
PgR,* n* (%)	Positive	98 (95.1)
	Negative	3 (4.9)
HER2,* n* (%)	Positive	7 (6.8)
	Negative	96 (93.2)
AR,* n* (%)	Positive	99 (96.1)
	Negative	4 (3.9)
PD-L1,* n* (%)	IC0	96 (93.2)
	IC1	6 (5.8)
	IC2	1 (1.0)
sTILs,* n* (%)	0–10%	80 (77.7)
	10–20%	17 (16.5)
	20–30%	4 (3.9)
	30–40%	2 (1.9)
Ki-67, mean (SD)		17.55 (18.17)

*IHC* immunohistochemistry, *ER* estrogen receptor, *PgR* progesterone receptor, *HER2* human epidermal growth factor 2, *AR* androgen receptor, *PD-L1* programmed death-ligand 1, *sTILs* stromal tumor-infiltrating lymphocytes, *SD* standard deviation

**Fig. 1:**
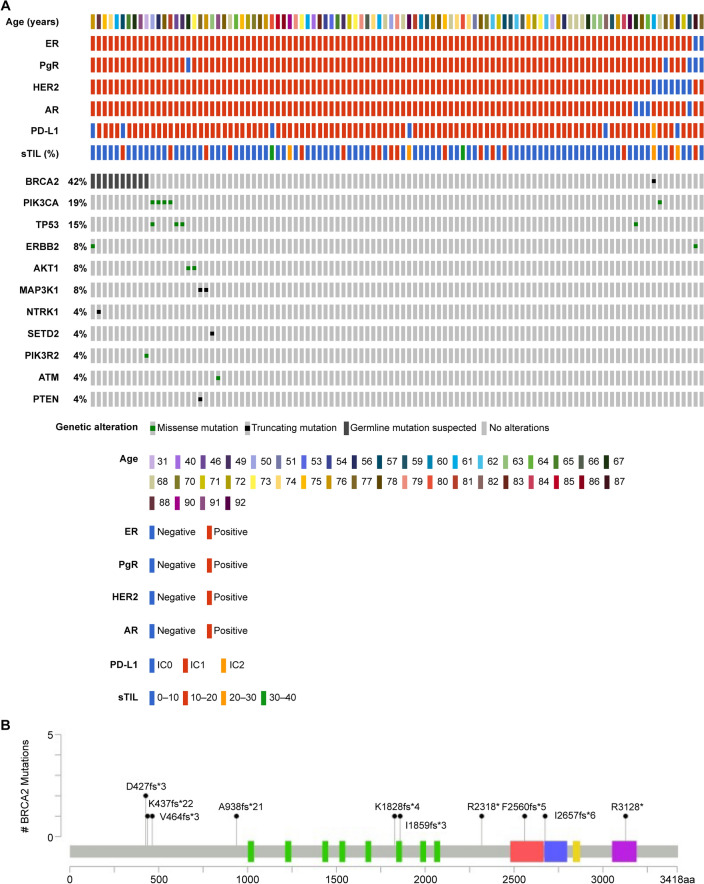
**A** Distribution of gene alterations in Japanese MBC patients; *SNV* single-nucleotide variant; **B** Locations of detected *BRCA2* mutations

Genetic alteration analysis using the NCC Oncopanel was performed on 87 samples from which sufficient DNA could be extracted. Various potentially pathogenic mutations were detected, including short insertions/deletions and splicing mutations; *BRCA2* was the gene most frequently affected (12.6% of total mutations). However, of the 11 *BRCA2* mutations observed, 10 (11.5% of total mutations) were considered germline variants based on VAF. Mutations were also detected in *PIK3CA* (5.7%); *TP53* (4.6%); *ERBB2*, *AKT1*, and *MAP3K1* (2.3% each); and *NTRK1*, *SETD2*, *PIK3R2*, *ATM*, and *PTEN* (1.1% each). One patient (1.1%) had homozygous deletions in *BRCA1* and *BRCA2*. Amplification was most frequent in *CCND1* (6.9%), followed by *FGFR1* and *MYC* (3.4%), then *ERBB2* and *GNAS* (2.3%) (Table 3, Fig. 1A). *BRCA2* mutation sites are shown in Fig. 1B.

**Table 3:** Gene alterations detected by NCC Oncopanel (*N* = 87)

		*n* (%)
Gene alterations,* n* (%)	Yes	31 (35.6)
	No	56 (64.4)
Indels, SNVs, splices,* n* (%)	*BRCA2*	11 (12.6)
	*PIK3CA*	5 (5.7)
	*TP53*	4 (4.6)
	*ERBB2*	2 (2.3)
	*AKT1*	2 (2.3)
	*MAP3K1*	2 (2.3)
	*NTRK1*	1 (1.1)
	*SETD2*	1 (1.1)
	*PIK3R2*	1 (1.1)
	*ATM*	1 (1.1)
	*PTEN*	1 (1.1)
Homozygous deletion,* n* (%)	*BRCA1*	1 (1.1)
	*BRCA2*	1 (1.1)
Amplification,* n* (%)	*CCND1*	6 (6.9)
	*FGFR1*	3 (3.4)
	*MYC*	2 (2.3)
	*ERBB2*	2 (2.3)
	*GNAS*	2 (2.3)

Indel, single-nucleotide insertion/deletion; *SNV* single-nucleotide variant

In addition, we aimed to analyze whether the *BRCA1* promoter region in our samples was methylated. However, only 27 samples had a sufficient number of DNA for the DNA methylation analysis and no methylation of the *BRCA1* promoter region was observed in any of the analyzed samples.

### Survival analysis

**Fig. 2:**
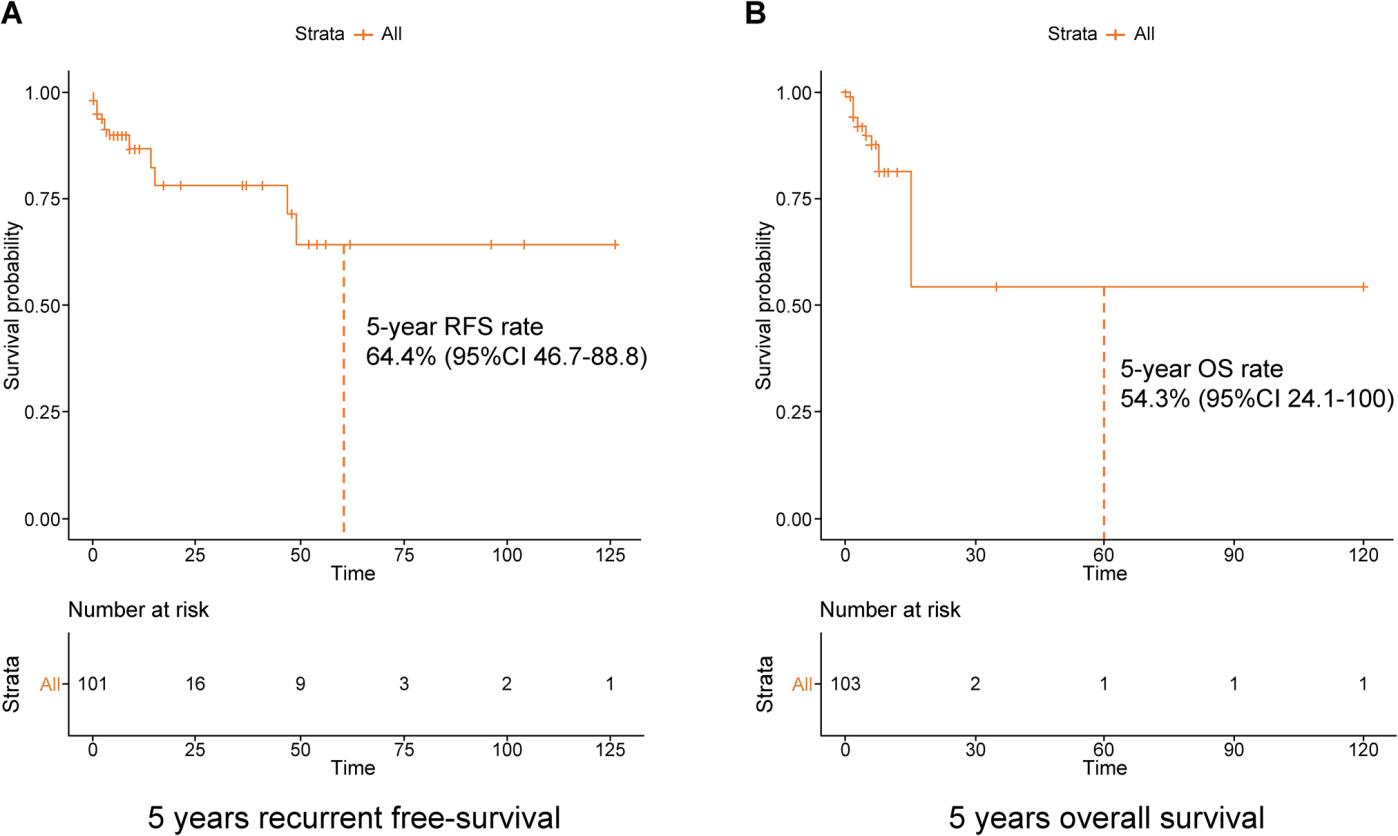
Survival analysis of Japanese MBC patients **A** Recurrence-free survival. **B** Overall survival. *RFS* recurrence-free survival, *OS* overall survival

Survival analysis showed a 5-year RFS rate of 64.4% (95% confidence interval [CI] 46.7–88.8) and a 5-year OS rate of 54.3% (95% CI 24.1–100.0) (Fig. 2). Figure S1 shows the RFS and OS of each MBC subtype. The median follow-up time for RFS and OS was 5 months and 4 months, respectively. Both RFS and OS tended to be worse in HR − /HER2 − cases, although the number of patients and follow-up time were insufficient to determine statistical significance.

## Discussion

To the best of our knowledge, this is the first comprehensive study of biomarkers in Japanese patients with MBC. We retrospectively analyzed samples from 103 patients with MBC and compared their clinicopathological characteristics. As in previous reports from Western countries [21], the median age of patients with MBC in our study (71 years) was higher than that of patients with FBC [22, 23]. In Western countries, MBC is usually detected at a more advanced stage, with half of the cases presenting with distant metastasis at the time of diagnosis [5, 8, 9], and the preferred first-line treatment being mastectomy together with adjuvant therapy.

Most MBC cases in our study were node negative, which could be due to the lower average body mass of Japanese patients or due to early detection. Nevertheless, most patients underwent a mastectomy. Breast-conserving surgery may be technically difficult in Japanese patients because of their smaller average body size. The BMI and lymph node metastasis are correlated in FBC [24]. There is no screening program to detect MBC. As males have relatively smaller breasts compared to females, it is easier to notice the changes in breast morphology. However, in obese patients, it is difficult to detect small tumors as the tumor burrows into the fat tissues. Axillary lymph node or sentinel node biopsy was performed in 90.5% of Japanese MBC cases, which is significantly higher than the rate in the US and Europe (76.4%). Comorbidities were reported in the majority of patients (63.7%), which may be linked to their advanced age. Among patients with MBC, comorbidities are also more common in the elderly [25].

The largest analysis to date of men with breast cancer, including more than 16,000 men, found a 5-years OS of 45.8%, much lower than that observed for women (60.4%) [10]. In this study, 5-year RFS was 64.4%, and 5-years OS was 54.3%. The median follow-up time for RFS was 5 months and that for OS was 4 months.

Like patients in Western countries, Japanese patients with MBC exhibited a high rate of HR positivity. The rate of AR positivity was significant and higher than that reported in previous studies [21, 26]. In contrast, most Japanese patients with MBC did not express PD-L1 (93.2%). For comparison, approximately 40% of triple-negative FBC patients are reported to be positive for PD-L1. MBC cases in this study were mostly ER-positive, which may be a factor in part to the low proportion of sTILs in these cases (less than 20% in 94.2% of cases). TILs are thought to affect the response to chemotherapy, HER2-targeted therapy [27], and pharmacotherapy even in HR + patients [16] and maybe a factor in determining the appropriate treatment in MBC.

The most frequently altered gene in our study was *BRCA2*. Piscuoglio et al. conducted a relatively large genomic analysis of MBC and reported a high frequency of alterations in *PIK3CA*, *GATA3*, *TP53*, and *MAP3K1*, similar to FBC [28]. In the present study, alterations in *PIK3CA*, *TP53*, and *MAP3K1* were detected relatively frequently, with a similar trend.

Methylation of the *BRCA1* promoter region is known to be one of the causes of breast cancer development in women [29] and has been reported as an oncogenic mechanism in familial breast cancer [30], but it was not detected in this study. It is possible that methylation of the *BRCA1* promoter region does not contribute to carcinogenesis in Japanese patients with MBC; however, due to the small number of samples analyzed in this study, we cannot draw such a conclusion with certainty. Methylation of *RAD51B* and *XRCC3* has been reported to contribute to carcinogenesis [26] and should be the subject of further investigation.

Most of the *BRCA2* variants detected in this study were presumed to be germline variants based on VAF. In a previous gene mutation analysis conducted in tumor tissue alone, more than 80% of *BRCA1* and *BRCA2* variants detected were reported to be of germline origin [31]. Therefore, based on our data, approximately 12% of Japanese MBC cases were suspected to have hereditary breast and ovarian cancer (HBOC). In addition, germline mutations in *BRCA2* have been shown to increase the risk of MBC by 80–100 times [32]. A family history of cancer was reported by 49.5% of participants in our study, which is relatively low. The low reporting rate could be due to a lack of knowledge and focus on HBOC among oncologists prior to the introduction of its guidelines in 2017. Among patients with a family history of HBOC or related cancers, 23.3% reported a history of breast cancer, 3.9% reported pancreatic cancer, 1.0% reported prostate cancer, and none reported a family history of ovarian cancer. In addition, more patients reported a family history of breast cancer than in previous reports [2]. This bias may be due to the fact that the most common causative gene was *BRCA2*, which causes HR + breast cancer, pancreatic cancer, and prostate cancer, as opposed to *BRCA1*, which causes triple-negative breast cancer and ovarian cancer. As stated in the National Comprehensive Cancer Network guidelines [33], genetic testing should be discussed with MBC patients in order to account for HBOC.

The limitations of this study include the retrospective nature of the study and the small number of cases due to the rarity of the disease. Unfortunately, the low number of samples and the poor quality of old specimens did not permit accurate detection of DNA methylation levels. In future, a large-scale registry and tissue bank should be constructed to study the biology of rare cancers and rare mutations, which will facilitate the development of novel treatments. In addition, MBC patients should be included in prospective breast cancer trials so clinical and biological data can be accumulated. Also, we did not collect the information about treatment efficacy, due to difficulties in assessing treatment responses in adjuvant settings.

In conclusion, this is the first comprehensive biomarker study of Japanese patients with MBC. Due to the observed clinicopathological differences between MBC in Western countries and Japan, further prospective studies are needed to evaluate the most suitable treatment strategies for MBC.

## Supplementary Information

Below is the link to the electronic supplementary material.

Supplementary file1 (DOCX 145 KB)

## Data Availability

The datasets generated during and/or analyzed during the current study are not publicly available because they are held by National Cancer Center Research Institute but are available from the corresponding author on reasonable request.
