# Supplementary material for: Clinicopathological features, genetic alterations, and BRCA1 promoter methylation in Japanese male patients with breast cancer
Source: Breast Cancer Res Treat. 2022 Dec 9;197(3):593–602. doi: 10.1007/s10549-022-06822-x (PMC9883318; doi:10.1007/s10549-022-06822-x)
Supplement: Supplementary file 1 — Supplementary file1 (DOCX 145 KB) [file 10549_2022_6822_MOESM1_ESM.docx]

**Supplementary Information**

**Journal name:** *Breast Cancer Research and Treatment*

**Clinicopathological features, genetic alterations and BRCA1 promoter methylation in Japanese male patients with breast cancer**

Akihiko Shimomura^1^*, Masayuki Yoshida^2^, Takashi Kubo^3^, Satoshi Yamashita^4^, Emi Noguchi^5^, Aiko Nagayama^6^, Toru Hanamura^7^, Miki Okazaki^8^, Toru Mukohara^9^, Asako Tsuruga^10^, Kiyo Tanaka^11^, Yukino Kawamura^1,12^, Toru Higuchi^13^, Yoko Takahashi^14^, Sasagu Kurozumi^15^, Tetsu Hayasida^6^, Hitoshi Ichikawa^3^, Toshikazu Ushijima^4^, Akihiko Suto^16^

^1^Department of Breast and Medical Oncology, National Center for Global Health and Medicine, Tokyo, Japan

^2^Department of Pathology, National Cancer Center Hospital, Tokyo, Japan

^3^Department of Clinical Genomics, National Cancer Center Research Institute, Tokyo, Japan

^4^Division of Epigenomics, National Cancer Center Research Institute, Tokyo, Japan

^5^Department of Medical Oncology, National Cancer Center Hospital, Tokyo, Japan

^6^Department of Surgery, Keio University School of Medicine, Tokyo, Japan

^7^Department of Breast Oncology, Tokai University School of Medicine, Kanagawa, Japan

^8^Department of Breast Oncology, Tokyo Medical University, Tokyo, Japan

^9^Department of Medical Oncology, National Cancer Center Hospital East, Kashiwa, Japan

^10^Division of Breast Surgical Oncology, Department of Surgery, Showa University Hospital, Tokyo, Japan

^11^Department of Breast and Endocrine Surgery, Toranomon Hospital, Tokyo, Japan

^12^National Center for Global Health and Medicine Research Course in Advanced Medical Specialties, Juntendo University Cooperative Graduate School, Tokyo, Japan

^13^Breast Surgery Unit, Japan Red Cross Saitama Hospital, Saitama, Japan

^14^Department of Breast Surgical Oncology, Cancer Institute Hospital of Japanese Foundation for Cancer Research, Tokyo, Japan

^15^Department of Breast Surgery, International University of Health and Welfare, Chiba, Japan

^16^Department of Breast Surgery, National Cancer Center Hospital, Tokyo, Japan

***Corresponding author**: Akihiko Shimomura, MD, PhD; E-mail: akshimomura@hosp.ncgm.go.jp


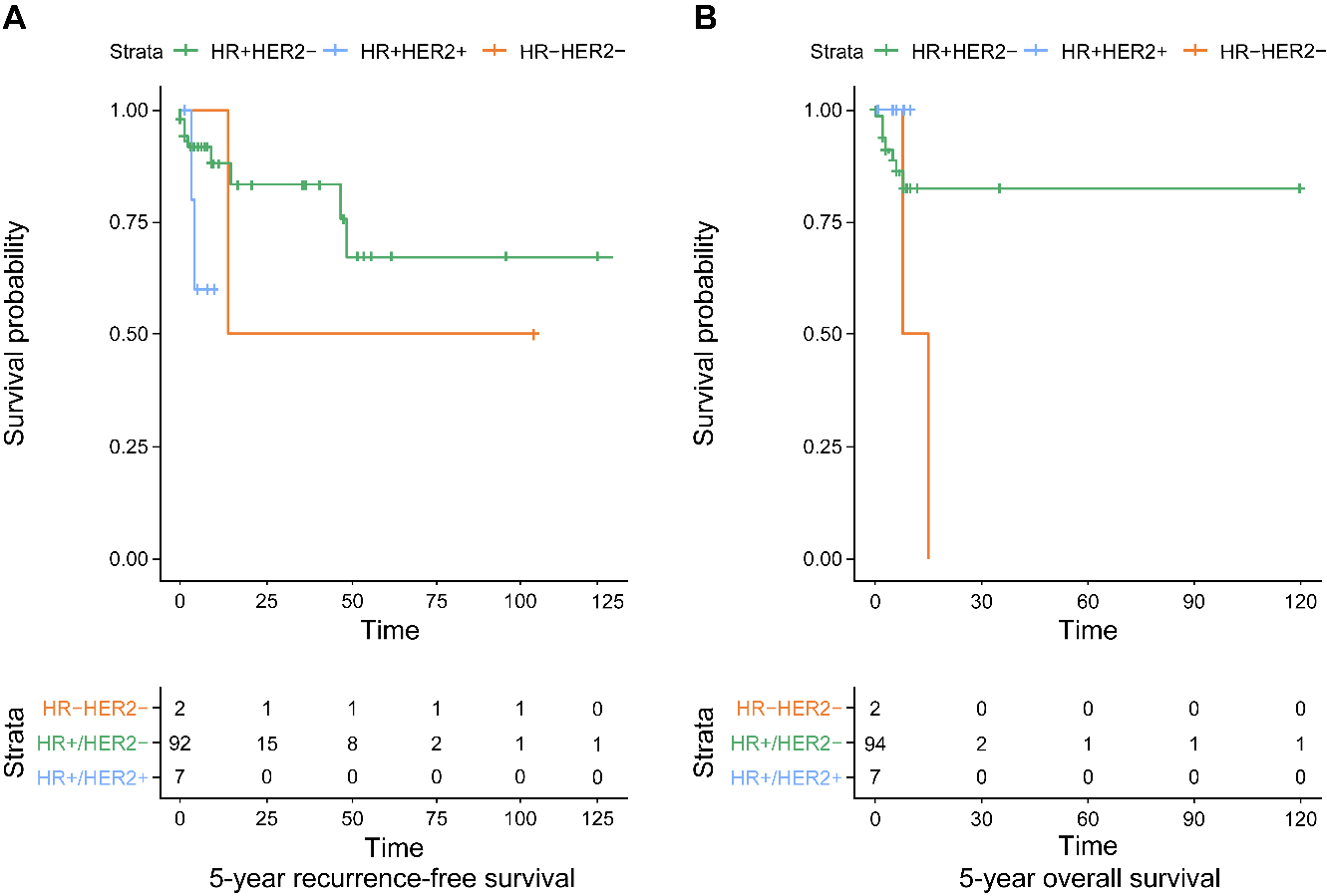


**Fig. S1** Survival analysis of Japanese male breast cancer patients by cancer subtype

(A) Recurrence-free survival. (B) Overall survival.

**Table S1** Immunohistochemical staining of FFPE specimens

| Primary antibody | Clone | Supplier | Pretreatment | Dilution | Detection | Platform |
| --- | --- | --- | --- | --- | --- | --- |
| ER | SP1 | Roche | CC1 | Prediluted | UltraView | VENTANA BenchMark XT |
| PgR | 1E2 | Roche | CC1 | Prediluted | UltraView | VENTANA BenchMark XT |
| HER2 | 4B5 | Roche | CC1 | Prediluted | UltraView | VENTANA BenchMark XT |
| AR | AR441 | DAKO | TRS pH9 | 1/50 | Envision | DAKO Autostainer Link 48 |
| PD-L1 | SP142 | Roche | CC1 | Prediluted | OptiView | VENTANA BenchMark XT |

**Table S2** Details of comorbid malignancies (*N* = 103)

| Cancer | *n* (%) |
| --- | --- |
| Gastric | 8 (7.8) |
| Colorectal | 7 (6.8) |
| Prostate | 7 (6.8) |
| Esophageal | 3 (2.9) |
| Laryngeal | 2 (1.9) |
| Thymic | 1 (1.0) |
| Thyroid | 1 (1.0) |
| NET | 1 (1.0) |
| Bladder | 1 (1.0) |
| Oral cavity | 1 (1.0) |
| Breast | 1 (1.0) |
| HCC | 1 (1.0) |
| Lung | 1 (1.0) |
| Skin | 1 (1.0) |
| Others | 4 (3.9) |

NET, neuroendocrine tumor; HCC, hepatocellular carcinoma

Table S3.

| SNV&Indel | **Sample** | **GENE** | **CDS_CHANGE** | **AA_CHANGE** |
| --- | --- | --- | --- | --- |
|  | 1 | BRCA2 | C9382T | R3128* |
|  | 1 | ERBB2 | G2033A | R678Q |
|  | 2 | AKT1 | G49A | E17K |
|  | 3 | BRCA2 | 1274_1274delA | D427fs*3 |
|  | 4 | NTRK1 | 1660_1660delC | R554fs*104 |
|  | 4 | BRCA2 | 7677_7678delTT | F2560fs*5 |
|  | 5 | SETD2 | T7533+2C | _ |
|  | 6 | BRCA2 | 2806_2809delAAAC | A938fs*21 |
|  | 6 | PIK3R2 | G1669T | D557Y |
|  | 7 | TP53 | G31C | E11Q |
|  | 8 | BRCA2 | 8015_8016insA | I2675fs*6 |
|  | 9 | ATM | C1009T | R337C |
|  | 10 | BRCA2 | C6952T | R2318* |
|  | 11 | BRCA2 | 5574_5577delAATT | I1859fs*3 |
|  | 12 | PIK3CA | G1633A | E545K |
|  | 13 | BRCA2 | 1301_1304delAAAG | K437fs*22 |
|  | 14 | PIK3CA | A331G | K111E |
|  | 15 | BRCA2 | 1389_1390delAG | V464fs*3 |
|  | 16 | TP53 | G91A | V31I |
|  | 17 | PIK3CA | A3140G | H1047R |
|  | 18 | BRCA2 | 5479_5483delATTAA | K1828fs*4 |
|  | 19 | PIK3CA | A3140G | H1047R |
|  | 20 | ERBB2 | C929T | S310F |
|  | 20 | ERBB2 | G2329T | V777L |
|  | 21 | AKT1 | G49A | E17K |
|  | 22 | MAP3K1 | 3230_3231insA | N1079fs*2 |
|  | 23 | PIK3CA | A3140G | H1047R |
|  | 23 | TP53 | G91A | V31I |
|  | 24 | TP53 | G31C | E11Q |
|  | 25 | BRCA2 | 1274_1274delA | D427fs*3 |
|  | 26 | MAP3K1 | C3739T | Q1247* |
|  | 26 | PTEN | 861_863delinsTTTTT | E288* |
|  |  |  |  |  |
| CNV | **Sample** | **GENE** | **TYPE** | **CNV** |
|  | 3 | MYC | amplification | 4.25901851 |
|  | 16 | FGFR1 | amplification | 4.35451937 |
|  | 16 | CCND1 | amplification | 3.36757836 |
|  | 17 | CCND1 | amplification | 6.08738635 |
|  | 17 | ERBB2 | amplification | 10.9488044 |
|  | 21 | CCND1 | amplification | 5.25237164 |
|  | 21 | GNAS | amplification | 5.24567345 |
|  | 27 | BRCA1 | expnic homozygous deletion | 0.092312131 |
|  | 28 | FGFR1 | amplification | 7.08810512 |
|  | 28 | CCND1 | amplification | 6.6534195 |
|  | 29 | CCND1 | amplification | 3.055404662 |
|  | 30 | MYC | amplification | 4.7493523 |
|  | 30 | ERBB2 | amplification | 5.25707013 |
|  | 31 | FGFR1 | amplification | 5.78802568 |
|  | 31 | CCND1 | amplification | 3.377776104 |
|  | 31 | GNAS | amplification | 4.00650367 |
|  | 32 | CCND1 | amplification | 8.12055313 |
